# Pre-treatment assay of 5-fluorouracil degradation rate (5-FUDR) to improve prediction of 5-fluorouracil toxicity in gastro-esophageal cancer

**DOI:** 10.18632/oncotarget.12571

**Published:** 2016-10-11

**Authors:** Marina Borro, Andrea Botticelli, Federica Mazzuca, Elisa Concetta Onesti, Giovanna Gentile, Adriana Romiti, Bruna Cerbelli, Eva Mazzotti, Luca Marchetti, Luana Lionetto, Maurizio Simmaco, Paolo Marchetti

**Affiliations:** ^1^ Department of Neurosciences, Mental Health and Sensory Organs (NESMOS), Sapienza University of Rome, Rome, Italy; ^2^ Advanced Molecular Diagnostic, IDI-IRCCS, Rome, Italy; ^3^ Department of Clinical and Molecular Medicine, Sapienza University of Rome, Rome, Italy; ^4^ Department of Radiological Oncological and Pathological Sciences, Sapienza University of Rome, Rome, Italy; ^5^ Department of Clinical Oncology, Sapienza University of Rome, Rome, Italy

**Keywords:** 5-FU degradation rate, phenotypic test, 5-FU toxicity, gastro-esophageal cancer, DPYD

## Abstract

**Background:**

5-fluorouracil (5-FU) based chemotherapy is the most common first line regimen used in gastric and gastroesophageal junction cancer, but development of severe toxicity is a main concern in the treatment. The present study is aimed to evaluate a novel pre-treatment assay, known as the 5-FU degradation rate (5-FUDR), as a predictive factor for 5-FU toxicity.

**Methods:**

Pre-treatment 5-FUDR and gene polymorphisms related to 5-FU metabolism (*DPYD*IVS14+1G>A, *MTHFR*A1298T or C677T, *TMYS* TSER) were characterized in gastro-esophageal cancer patients. Association with toxicities was retrospectively evaluated, using multivariate logistic regression analysis.

**Results:**

107 gastro-esophageal cancer patients were retrospectively analyzed. No relation between gene polymorphisms and toxicity were detected, while low (< 5^th^ centile) and high (> 95^th^ centile) 5-FUDRs were associated with development of grade 3-4 toxicity (OR 11.14, 95% CI 1.09-113.77 and OR 9.63, 95% CI 1.70-54.55, *p* = 0.002).

**Conclusions:**

Compared to currently used genetic tests, the pre-treatment 5-FUDR seems useful in identifying patients at risk of developing toxicity.

## INTRODUCTION

5-Fluorouracil (5-FU) and its pro-drug capecitabine, alone or in combination with epirubicin, oxaliplatin, irinotecan, represent the most used chemotherapy treatments of gastroesophageal cancer, in both the adjuvant and palliative settings. [1–2]

Despite the benefit of fluoropyrimidine treatment, the development of severe toxicities often lead to dose reduction, delaying of administration and therapy discontinuation. The most common side effects associated with 5-FU are diarrhea, mucositis, myelosuppression, hand foot syndrome and rarely cardiac toxicity. [3] Grade 3 or 4 toxicities are reported in about 30% of patients, with a mortality rate of 0.5 %. [3, 4] The efforts of establishing effective tests to identify such toxicities preemptively led to the development of genotyping or phenotyping methods, in order to evaluate the efficiency of the individual 5-FU metabolism. [5, 6]

Inside the cell, 5-FU is transformed by different enzymes in both active and inactive metabolites. The balance between inactive metabolites and therapeutic metabolites is thought to be the basis of the inter-individual differences in toxicity and efficacy of 5-FU based treatments. [7]

The dihydropyrimidine dehydrogenase enzyme (DPD), encoded by the *DPYD* gene, inactivates about 80% of the administrated 5-FU, by transforming it into 5,6-dihydro-5-fluorouracil. *DPYD* has been the top candidate for pharmacogenetic studies on 5-FU toxicity, as a reduced DPD activity results in an increased half-life of the drug, and thus an increased risk of toxicity. [5, 8–14] The splice site variant IVS14+1G>A polymorphism in the *DPYD* gene (rs3918290; allele A also known as *2A allele) is the most consistent genetic marker for toxicity. Unfortunately the low minor allele frequency and the fact that just about a 50% of the *2A allele carriers actually develop severe toxicity limit its prediction power. [6] In a recent published study, conducted on more than 2000 patients, DPYD*2A polymorphic cases were treated with a 50% reduced dose of fluoropyrimidine. The results showed a significant reduction of severe toxicity from 73% to 28% and with 0% toxic deaths in polymorphic allele carriers. [15] However, considering the low frequency of DPYD polymorphic allele in general population [16], this method could lead to the identification of only about 1% of patients at risk of developing severe toxicity. DPYD polymorphism is frequently assessed in patients eligible for 5-FU treatment together with the C677T and the A1298T polymorphisms in the *MTHFR* gene and with the *TSER* polymorphism in the TS gene. In fact,the main mechanism of the 5-FU action consists of inhibition of thymidylate synthase (TS) through the active metabolite, fluorodeoxyuridine monophosphate (FdUMP), which forms an inactive ternary complex with TS and 5-10-methylenetetrahydrofolate (MTHF). Optimal inhibition of TS requires an elevated level of MTHF, which is regulated by the methylenetetrahydrofolate reductase enzyme (MTHFR). [7] As a consequence, polymorphisms affecting TS and MTHFR levels are presumed to be determinants of 5-FU clinical response, but indeed their clinical utility is still controversial. [17–26]

The phenotypic tests available for preemptive evaluation of risk for severe toxicity are generally less diffused compared to pharmacogenetics, even if they could be potentially more effective in identifying patients at risk. However, most of such tests are limited to detection of DPD activity, not considering possible alteration in other 5-FU metabolic enzymes and eventually in 5-FU transporters. [7, 27, 28] To overcome this limit, we have previously developed a pre-treatment *ex-vivo* assay to determine the velocity at which the peripheral blood mononuclear cells (PBMC) metabolize 5-FU. [29] This parameter, named individual 5-FU degradation rate (5-FUDR, expressed as nmol of drug consumed by cells in a time unit), is performed in intact and viable cells, thus it the final result of all the enzymatic transformation of 5-FU, not just the DPD activity. The individual, pre-treatment 5-FUDR value, was found to be significantly lower in patients who develop grade 3-4 toxicity.[29]

The Oncology Unit of the Sant'Andrea Hospital of Rome adopted the pre-treatment 5-FUDR as a routine test giving a “toxicity warning” to plan careful monitoring of patients with a low 5-FUDR value. In general population, the 5-FUDR is a continuous parameter with a normal distribution (mean value 1.54 ± 0.41 ng 5-FU/ml/10^6^ cells/min), whereas the mean 5-FUDR value in carriers of the *DPYD* *2 allele is 0.81±0.29 ng 5-FU/ml/10^6^ cells/min. [30] We have recently showed that a significant reduction of the individual 5-FUDR value is also found in subject carriers of a *DPYD* haplotype involving three polymorphisms apart from the *2. [31] Moreover, 5FU-DR value seems to be related to severe adverse events in colorectal cancer patients, with a higher toxicity rate when 5-FU degradation is slowed (5-FUDR ≤0.85 ng/ml/10^6^ cells/min) or accelerated ( 5-FUDR ≥ 2.2 ng/ml/10^6^ cells/min), regardless of the DPYD status. [30] Since low 5-FUDR value was also found in subjects who were non carriers of defective *DPYD* alleles, we hypothesized that it could identify a further fraction of patients who will likely develop severe 5-FU toxicity.

The present study investigated the association between individual 5-FUDR, polymorphisms in *DPYD*, *MTHFR,TSER* and toxicity in a population of 107 gastric and gastro-esophageal junction cancer patients.

## PATIENTS AND METHODS

### Patients

Patients, with a histological confirmed diagnosis of gastric and gastro-esophageal junction cancer, who had been undergoing chemotherapy at the Sant'Andrea Hospital of Rome in the period 2009-2012, were enrolled in this retrospective study.

The inclusion criteria were: patients with measurable disease, adequate organ function and performance status of grade 0, 1 or 2 as defined by the Eastern Cooperative Oncology Group [33]; patients who had undergone 5-FU based chemotherapy (DCF, EOX, FOLFOX, XELOX, FOLFIRI); patients who had undergone pre-treatment assay of 5-FUDR and characterization of polymorphisms of *MTHFR*, *TSER* and *DPYD* genes. Exclusion criteria were: relevant diseases within 6 months (i.e.: myocardial infarction, lung fibrosis, etc) and 5-FU based chemotherapy in the past.

Chemotherapy cycles were administered every 2 or 3 weeks according to the scheme. All toxicities were graded according to the National Cancer Institute Common Terminology Criteria for Adverse Event version 3 (CTCAE 3.0) and toxicity assessments performed at day 1 of each cycle until the end of treatment. [33]

The study was conducted in accordance with the Declaration of Helsinki and the protocol was approved by the institutional ethic committee.

### Genotyping

To analyze germinal polymorphisms genomic DNA was isolated from peripheral blood, by mean of the X-tractor Gene system (Corbett Life Science, Australia). The commercial kit for fluoropyrimidine response (Diatech, Jesi, Italy) was used, according to the manufacturer's protocol, to analyze the following splice-site polymorphisms: IVS14+1G>A in the *DPYD* gene and C677T and A1298C SNPs in MTHFR gene. Briefly by using PCR with specific primers, the region covering the SNP of interest was amplified. Subsequently it was sequenced using the Pyrosequencer PyroMark ID system (Biotage AB and Biosystems, Uppsala, Sweden). PCR (fluoropyrimidine response - Diatech, Jesi, Italy) was used also to determine the variable number of tandem repeats (VNTR; 2R or 3R) in the thymidylate synthase enhancer region (TSER), visualized onto 2,2% agarose gel.

### Determination of the individual 5-FU degradation rate

The assay for 5-FUDR has been established in the Sant'Andrea Hospital of Rome as a routine clinical analysis prior to fluorouracil-based chemotherapies and is carried out following medical prescription. The test is performed, as previously reported [29], using a 5-FUDR assay kit (Eureka srl-Lab Division, Chiravalle, Ancona, Italy) with a HPLC-MS/MS instrument including an Agilent 1100 chromatographic system coupled to an API 3200 triple quadrupole (ABSCIEX, Framingham, MA, USA). Freshly prepared peripheral blood mononuclear cells (2.5-3.5 × 10^6^ cells) are incubate with a known dose of 5-FU at 37°C, with shaking Cells aliquots are analyzed at time 0, 1 h and 2 h. Cells were lysed and centrifuged. 5-FU concentration in the supernatants is quantified by HPLC-MS/MS and the 5-FUDR is expressed as ng 5-FU/ml/10^6^ cells/min. [29]

### Statistical analysis

STATA software, version 11.0 (StataCorp, College Station, Tex) was used for statistical analysis Data are presented as mean ± standard deviation (SD). Patients were categorized by sex, age (<=median age, >median age), toxicity (grade 0-2, grade3-4), 5-FUDR value.

In a previous published study we analyzed the continuous variable 5-FUDR on 1010 cancer patients, before receiving fluoropyrimidine treatment. [30] Patients were classified into the three following metabolic classes, according to the values of the 5^th^ and 95^th^ centile as determined by the normal distribution of 5-FUDR: poor metabolizers (PM; i.e. ≤ 5^th^ centile, ≤0.85 ng/ml/10^6^ cells/min); normal metabolizers (NM; i.e. > 5^th^ centile and < 95^th^ centile, > 0.85 ng/ml/10^6^ cells/min and < 2.2 ng/ml/10^6^ cells/min); ultra-rapid metabolizers (UM; i.e. ≥ 95^th^ centile, ≥ 2.2 ng/ml/10^6^ cells/min).

Chi-squared or Fisher exact test were used to establish differences between groups, as appropriate. Logistic regression models were useful for univariate and multivariate odds ratios (ORs) with associated 95% confidence intervals (CI) for variables associated with severe toxicities.

Test for deviation of polymorphisms’ distributions from the Hardy-Weinberg (HW) equilibrium was performed using the SNP Stats software. [34]

## RESULTS

We analyzed gene polymorphisms related to 5-FU response and the pretreatment 5-FUDR in 107 gastro-esophageal cancer patients (71 males, median age 68/69 years; 36 females, median age 64/65 years) Table 1. Major adverse events (CTC-grade 3 or 4) were encountered in 29 patients (27.1 %). The distributions of the analyzed gene polymorphisms (Table 2) were in Hardy-Weinberg equilibrium. The *DPYD* *2 allele was detected in just one heterozygous carrier, corresponding with the 1.28% frequency reported for the overall Italian population [31], hence this polymorphism has not been further considered in the analysis. However, this patient had a 5-FUDR below the 5^th^ centile (0.58 ng/ml/10^6^ cells/min) and developed a high grade toxicity. In the total samples analyzed, the 5-FUDR has a mean value of 1.61 ± 0.42 ng/ml/10^6^ cells/min, and is not significantly affected by age, gender, *MTHFR* A1298T or C677T polymorphisms nor by the TSER polymorphism (Table 1).

**Table 1:** Patients’ characteristics

		Number of patients	%
Sex	***Male***	71	66.36
	***Female***	36	36.64
Age category *	***≤ median***	56	52.34
	***>median***	51	47.66
Site of primary	***Gastro-oesophageal junction***	11	10.28
	***Gastric***	96	89.72
Stage	***Locally advanced***	49	45.79
	***Metastatic***	58	54.20
Type of treatment	***5-FU based***	59	55.14
	***Capecitabine based***	10	9.35
	***Monotherapy***	38	35.51

*for males 68/69yrs; for females 64/65yrs.

**Table 2:** 5-FUDR descriptive statistics by demographic and genetic characteristics (*N* = 107)

	Total		5-FUDR (mean±SD)	p*
	*N*	%		
**Sex** males females	71 36	66.36 33.64	1.60±0.43 1.63±0.42	0.762
Age category** <=median >median	56 51	52.34 47.66	1.64±0.43 1.58±0.42	0.458
***MTHFR A1298C*** AA AC CC	47 54 5	44.34 50.94 4.72	1.63±0.47 1.62±0.39 1.33±0.13	0.306
***MTHFR C677T*** CC CT TT	28 53 26	26.17 49.53 24.30	1.53±0.40 1.65±0.39 1.61±0.51	0.458
***TMYS TSER*** 2R2R 2R3R 3R3R	28 50 28	26.42 47.17 26.42	1.70±0.34 1.57±0.47 1.59±0.41	0.431

**Chi squared test or Fisher exact test; **for males 68/69yrs; for females 64/65yrs.

Table 3 reports the toxicities. Table 4 reports the distribution of low toxicity (grade 0-2) and severe toxicity (grade 3-4) among patients’ groups. Whereas neither sex, age categories, nor *MTHFR* and *TSER* genotype affect the development of higher grade toxicity. The 5-FUDR value is associated with the development of severe 5-FU toxicities. In particular, a significant increase (p=0.002) in the proportion of severe toxicities has been detected in both the patients’ group with a 5-FUDR poor metabolizers and for the patients’ group with a 5-FUDR ultra-rapid metabolizers The ORs adjusted for age and sex were 11.14 (95%CI 1.09-113.77) for the low 5-FU metabolizers and 9.63 (95%CI 1.70-54.55) for the ultra-rapid 5-FU metabolizers.

**Table 3:** Toxicities

	G1-2 toxicity (N)	G1-2 toxicity (%/107 pts)	G3-4 toxicity (N)	G3-4 toxicity (%/107 pts)
Hematological	16	14.95	20	18.69
Gastrointestinal	23	21.50	8	7.48
HFS	1	0.93	1	0.93
Other	15	14.02	2	1.87

**Table 4:** Distribution of grade 0-2 and grade 3-4 toxicities according to demographics, genetics and 5-FUDR

	Total		Toxicity Grade 0-2		Toxicity Grade 3-4		*p*	OR (95% CI)*	OR (95% CI)**
	*N*	%	*N*	%	*N*	%			
**Sex** males females	71 36	66.36 33.64	53 25	74.65 69.44	18 11	25.35 30.55	0.567	1 1.30 (0.53-3.15)	1 1.28 (0.50-3.32)
Age category*** <=median >median	56 51	52.34 47.66	42 36	75 70.59	14 15	25 29.41	0.608	1 1.25 (0.53-2.94)	1 1.47 (0.58-3.71)
***MTHFR A1298C*** AA AC CC	47 54 5	44.34 50.94 4.72	36 38 4	76.60 70.37 80	11 16 1	23.40 29.63 20	0.736	1 1.38 (0.56-3.37) 0.82 (0.08-8.10)	-
***MTHFR C677T*** CC CT TT	28 53 26	26.17 49.53 24.30	19 40 19	67.86 75.47 73.08	9 13 7	32.14 24.53 26.92	0.764	1 0.69 (0.25-1.88) 0.78 (0.24-2.52)	-
***TMYS TSER*** 2R2R 2R3R 3R3R	28 50 28	26.42 47.17 26.42	20 36 21	71.43 72 75	8 14 7	28.57 28 25	0.947	1 0.97 (0.35-2.71) 0.83 (0.25-2.73)	-
***5-FUDR*** <5th centile >5th≤95th >95th centile	4 96 7	3.74 89.72 6.54	1 75 2	25 78.13 28.57	3 21 5	75 21.88 71.43	0.002	1 10.71 (1.06-108.41) 8.93 (1.62-49.35)	1 11.14 (1.09-113.77) 9.63 (1.70-54.55)

* Crude odds ratio; **Odds ratio adjusted for age and gender; ***for males 68/69yrs; for females 64/65yrs.

## DISCUSSION

Due to the narrow therapeutic range of fluoropyrimidines, the ratio of the effective dose to toxic dose is small [35] and the risk of developing severe toxicity, with a small percentage of lethal events [3, 4], is a main concern for patients and oncologists. Despite the improvement led by the advent of pharmacogenetic screening for *DPYD*, the proportion of pre-emptive identification of patients at high risk of severe (grade G3-4) 5-FU toxicity is still inadequate. Against a 30% of grade 3-4 toxicities [3, 4], the *DPYD* polymorphisms identify about 1-3% of patients at risk, because of the low frequencies of specific alleles in the general population.[6, 31] Thus, we investigated the potential of the phenotypic test 5-FUDR to increase the detection of “high risk” patients prior to 5-FU administration, in order to plan careful monitoring of toxic effects and better manage the anti-cancer therapy.

Along with the normal distribution of the 5-FUDR value, two cut-off values associated with a significant higher risk for the onset of grade 3-4 toxicity were identified: the 5^th^and the 95^th^centiles (0.85 and 2.2 ng/ml/10^6^ cells/min, respectively). [30] In fact, in the analyzed cohort, subjects with a poor 5-FU metabolism present an 11.14 OR (95%CI 1.09-113.77) for grade 3-4 toxicity. The underlying toxicity mechanism in poor 5-FU metabolizers could be explained by decreased drug clearance, as also suggested by the association between low 5-FUDR values and the presence of defective *DPYD* alleles [5, 7, 9, 17], namely the *2A allele and the Hap7 haplotype [31]. However, this previous work showed that subjects who are carriers of normal *DPYD* alleles (concerning 15 analyzed SNPs) can anyway have a poor 5-FUDR. The present results support the hypothesis that, regardless the *DPYD* genotype, the 5-FUDR is a predictor of toxicities related to fluorouracil-based chemotherapies, and a parameter reflecting the overall fluoropyrimidine metabolism.

**Figure 1:**
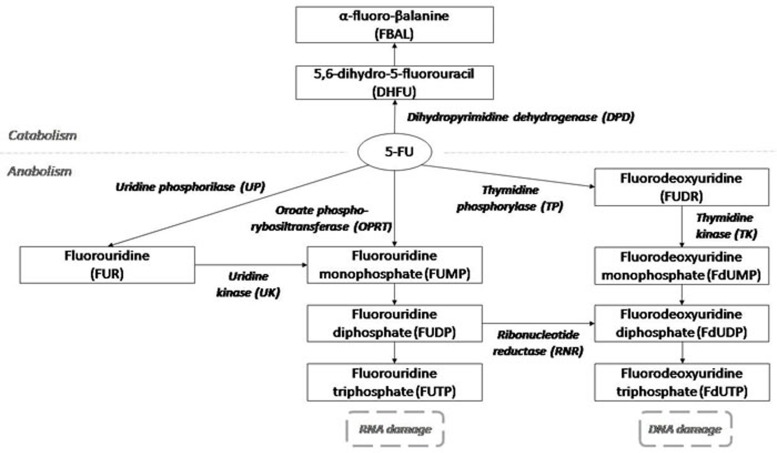
5-Fluorouracil metabolism

Interestingly, we also found an association between ultra-rapid (5-FUDR > 95^th^ centile) 5-FU metabolism (9.63 OR, 95%CI 1.70-54.55) with grade 3-4 toxicity. Theoretically, a high 5-FUDR could be due to an increased activity of the inactivating enzymes DPD, leading to a decline in the drug percentage transformed into active metabolites. However, a similar fast metabolism could derive by an increased activity of the 5-FU activating enzymes, leading to a raise in the concentration of therapeutic molecules. (Figure 1). Indeed, it has been demonstrated that the sensitivity to 5-FU is affected by polymorphisms in the orotate phosphoribosyltransferase gene (OPRT, transforming 5-FU in 5-fluorouridine monophosphate) and, in cancer tissues, by the level of activity of the OPRT enzyme and by the OPRT/DPD activities ratio. [36–39]

Since increased concentration of active metabolites could affect response as well as toxicity of the 5-FU treatment, it could be speculated that ultra-rapid 5-FU metabolizers could also have a different prognosis compared to non-ultrarapid metabolizers. This hypothesis is currently under investigation.

A limitation of our study is the enrollment of patients treated with combination therapy, even though to date the studies of associations between DPYD polymorphisms and 5-FU toxicities were based on 5-FU based chemotherapy instead of only 5-FU monotherapy.

Furthermore, in clinical practice only a few percentage of patients are treated with monotherapy so it's not easy to understand clear which toxicities depends on 5 -fluorouracil or on other drugs. However we presented at ESMO 2015 our results of patients treated with capecitabine monotherapy and it was confirmed the association between 5-FUDR classes and toxicity. [40]

The poor and ultra-rapid 5-FU metabolizer classes include by definition a 10% (< 5^th^ centile and > 95% centile) of the overall population. Thus, if used as a predictive factor, it has the potentiality to sensibly increase the identification of “at risk” patients, compared to pharmacogenetic testing. In the analyzed cohort of gastro-esophageal cancer patients, the 5-FUDR test classified 11 out of 107 subjects as patients with a consistent risk to develop grade 3-4 toxicity, of which 7 (63.6%) actually developed severe toxicity. The proportion of patients who developed severe toxicity identified preemptively by the 5-FUDR tests is 24.1% (7/29), a significant progress compared to the low percentage of toxicity potentially identifiable by the commonly used *DPYD* polymorphisms.

Considering that the 5-FUDR assay is a low-cost test (about 10 € per sample), it requires non-invasive sampling methods, and test results are available in one working day, it appears suitable and cost-effective for implementation in the routine pre-treatment panel of clinical evaluations.

Despite the limitations of the presented retrospective study, we observed appealing results. So, as future perspective, we highlight the importance of conducting prospective studies on larger sample size, on a homogeneous population in order to evaluate 5-FUDR impact on outcomes and with pharmacokinetics analysis on fluorouracil metabolites plasma concentration. More data on others cancer types, treated with fluoropyrimidine, are also auspicated.

## CONCLUSIONS

Compared to the available pharmacogenomic screening, the pre-treatment evaluation of 5-FUDR increases considerably the proportion of identified gastro-esophageal cancer patients at high risk for severe 5-FU toxicity, such as in colorectal cancer patients’ cohort preemptively.
